# MuscleSCS: a standardized programming framework combining neural neuromodulation and spinally mediated paraspinal muscle modulation

**DOI:** 10.3389/fpain.2026.1954865

**Published:** 2026-09-29

**Authors:** Matthias Hubert Morgalla

**Affiliations:** 1 INST-Institute for Neuromodulative Pain Therapy, Sigmaringen, Germany; 2 Medical Faculty, University of Tuebingen, Tuebingen, Germany

**Keywords:** alpha motoneuron modulation, chronic back pain, mechanotherapy, mechanotransduction, muscle modulation, muscleSCS, paraspinal muscles, spinal cord stimulation

## Abstract

**Background:**

Conventional spinal cord stimulation (SCS) primarily modulates neural pain-processing pathways but may not address persistent paraspinal muscle dysfunction associated with chronic back pain. MuscleSCS extends the therapeutic scope of implanted SCS systems by applying low-frequency stimulation through the existing epidural electrode to generate objectively verifiable paraspinal muscle activity. This activity is consistent with spinal motor pathway recruitment, although the specific neural pathways involved remain undetermined.

**Objective:**

To describe the conceptual basis, technical implementation, programming workflow, validation, anticipated results, and biological rationale of MuscleSCS as a method for combined neural and muscular neuromodulation.

**Methods:**

Low-frequency stimulation (typically 2–8 Hz, most commonly 4–8 Hz) is delivered through selected electrode contacts. Programs are optimized using contact screening, amplitude titration, patient feedback, visual and haptic assessment, surface electromyography, and muscle ultrasound. Programming is technically similar to conventional SCS and typically requires 15–20 min.

**Anticipated results:**

Previous clinical studies demonstrated technical feasibility, electrophysiologically verifiable muscle activation, ultrasound-confirmed activation of deep paraspinal muscles, and additional pain relief when MuscleSCS was combined with BurstDR SCS. The goal is comfortable muscle modulation rather than muscle strengthening.

**Conclusion:**

MuscleSCS provides a reproducible framework for spinally mediated muscle modulation that may complement conventional neural neuromodulation and improve pain relief. The underlying downstream mechanotransductive and immunomodulatory tissue responses provide a biologically plausible mechanistic framework derived from established skeletal-muscle mechanobiology but remain to be specifically validated for MuscleSCS.

## 1 Introduction

Chronic back pain is a heterogeneous condition in which nociceptive, neuropathic, degenerative, muscular, inflammatory, and psychosocial components may interact (1–3). In a subgroup of patients, paraspinal muscle dysfunction—including muscle insufficiency, altered recruitment, persistent protective guarding or increased muscle tone, local inflammatory processes, and disuse-related structural changes—may represent a clinically relevant contributor to persistent pain (4–10). However, the relationship between paraspinal muscle abnormalities and chronic low-back pain is heterogeneous, and these associations do not establish a causal role of muscle dysfunction in all patients. In patients with a clinically suspected muscular pain component, such abnormalities may contribute to residual pain despite otherwise successful neuromodulation of neural pain-processing pathways. This is clinically relevant because conventional SCS is primarily optimized for neural pain modulation, whereas a concomitant muscular component is generally addressed through pharmacological treatment, physiotherapy, or behavioral interventions (11). MuscleSCS was developed as a complementary approach for this selected clinical phenotype, rather than as a treatment for an assumed muscular pain generator in all patients with chronic back pain.

MuscleSCS uses the same epidural SCS electrode to generate low-frequency spinally mediated neuromuscular modulation (12). The neural structures initially recruited by MuscleSCS have not yet been specifically determined. Based on established neurophysiology of epidural spinal stimulation, motor output may involve activation of afferent fibers and trans-synaptic recruitment of spinal motor circuitry rather than direct stimulation of anterior-horn motoneurons. Regardless of the initial neural substrate, the resulting motor output ultimately involves spinal motoneurons and produces the clinically observed rhythmic paraspinal muscle activity. MuscleSCS is therefore described here as spinally mediated muscle modulation without assuming a specific direct recruitment mechanism.

The purpose of this article is to describe MuscleSCS as a reproducible method. The article therefore focuses on practical implementation, required materials, patient selection, programming procedure, validation methods, anticipated outcomes, limitations, troubleshooting, and the mechanobiological rationale. The central conceptual point is that MuscleSCS is not intended to strengthen muscles.

The directly observable effect of MuscleSCS is rhythmic stimulation-evoked paraspinal muscle activity. Repeated activation may be perceived by patients as muscle loosening or reduced tension and may potentially influence pathological muscular tone; however, a sustained reduction in resting muscle activity or stiffness has not yet been quantitatively demonstrated. Accordingly, reduction of pathological muscle tone and beneficial effects of repeated contraction-relaxation cycles are considered proposed therapeutic mechanisms rather than established physiological effects of MuscleSCS.

Repetitive, rhythmic muscle modulation may also create local mechanical stimuli capable of initiating mechanotransductive signaling cascades within dysfunctional paraspinal muscles (13).

Skeletal muscle is a clinically relevant pain-generating tissue (14, 15). Muscle nociceptors are located predominantly in connective tissue structures such as the perimysium and in the vicinity of vascular structures (14, 16, 17). They can be sensitized by inflammatory mediators, tissue hypoxia, bradykinin, serotonin, prostaglandins, adenosine triphosphate, histamine, nerve growth factor, substance P, and calcitonin gene-related peptide. Persistent inflammatory conditions in skeletal muscle may increase the density of neuropeptide-immunoreactive nerve endings, thereby increasing nociceptive input from the affected tissue (18, 19).

This pathophysiological model is particularly relevant for chronic back pain. Paraspinal muscles that remain in a state of chronic protective contraction may become painful, metabolically compromised, and increasingly sensitized (20–22). A therapeutic method capable of repeatedly reducing pathological muscle tone and inducing controlled mechanical tissue signals may therefore target a clinically meaningful pain generator that is not sufficiently addressed by conventional dorsal column SCS alone.

The term spinal cord stimulation is historically established and remains appropriate for the implanted technology. However, when describing MuscleSCS, the preferred term is modulation. This distinction is not merely semantic. MuscleSCS is not an attempt to generate maximal motor activation or muscle training; rather, it aims to generate controlled, comfortable, low-frequency neuromuscular modulation that can relax affected paraspinal muscles and provide pain relief. The same implanted device may therefore support two complementary therapeutic domains: conventional neural neuromodulation of pain pathways and muscle modulation mediated through spinal motor circuits.

Restorative neurostimulation of the lumbar multifidus represents a clinically established muscle-directed neuromodulation approach for selected patients with chronic mechanical low-back pain associated with impaired multifidus neuromuscular control (23, 24). By stimulating the medial branch of the dorsal ramus, the therapy aims to restore impaired multifidus activation and neuromuscular control of lumbar spinal stability rather than merely to strengthen the muscle. Recent five-year follow-up data from ReActiv8-B have demonstrated durable clinical benefit (24), and the 2025 RESTORE randomized trial further strengthened the evidence for this therapeutic concept (25). MuscleSCS is conceptually distinct because it uses an epidural SCS electrode to generate broader spinally mediated paraspinal muscle modulation rather than selectively targeting the multifidus motor pathway.

Although MuscleSCS generates rhythmic muscle activation, its therapeutic objective is not sustained contraction or strengthening. Rather, repeated low-frequency cycles of contraction and relaxation are intended to interrupt persistent tonic muscle activity and promote a more physiological pattern of muscle activation. Secondary adaptive or regenerative tissue effects resulting from repeated mechanobiological stimulation cannot be excluded and require further investigation.

These approaches should therefore be regarded as complementary rather than competing. Restorative multifidus neuromodulation aims to improve segmental stability by strengthening a key stabilizing muscle (26, 27). MuscleSCS aims to engage spinal motor pathways, resulting in spinally mediated modulation of paraspinal muscle activity and tone through an existing SCS system. Both methods are muscle-directed but operate through different anatomical access routes, therapeutic objectives, and biological mechanisms.

Accordingly, the novel contribution of the present Methods Article is not a new clinical efficacy dataset or the discovery of epidural motor recruitment, but the formalization of MuscleSCS into a standardized, technically specified, and prospectively testable programming framework that integrates previously reported clinical observations with explicit procedures for patient selection, stimulation programming, response confirmation, safety, and follow-up.

## 2 Conceptual basis of MuscleSCS

MuscleSCS expands the therapeutic concept of SCS from pure neural neuromodulation toward combined neural and muscular neuromodulation. In conventional SCS, electrical fields are programmed primarily to modulate dorsal column pathways and alter pain transmission (28–31). In MuscleSCS, low-frequency programs are used to engage spinal motor circuits, in order to generate rhythmic paraspinally mediated muscle modulation. The patient experiences this as a comfortable, rhythmic muscle movement or deep muscle activity rather than paresthesia alone.

The proposed conceptual sequence is: epidural SCS electrode → low-frequency spinal modulation → spinally mediated motor output → rhythmic paraspinal muscle activity → proposed modulation of muscle tension and mechanical tissue signaling → hypothesized downstream mechanotransductive and immunomodulatory effects → potential reduction in nociceptor sensitization and pain.

The same implanted electrode thus becomes a dual-purpose interface. It continues to provide established neural neuromodulation when programmed for conventional SCS waveforms and can also provide spinally mediated muscle modulation when programmed in a low-frequency MuscleSCS mode. This dual use is clinically attractive because no additional implanted electrode or peripheral nerve lead is required in patients with an existing compatible SCS system.

## 3 Materials and equipment

The following materials and equipment are required or optional for MuscleSCS implementation and validation (Table 1).

**Table 1:** Required equipment and optional confirmation tools for MuscleSCS programming.

Category	Required or optional	Purpose
Implanted SCS system with epidural lead	Required	Delivery of conventional SCS and low-frequency MuscleSCS programs through the same electrode
Percutaneous paddle lead or octrode	Required	Segmental spinal access; both lead types have been used clinically
Programming device and clinician programmer	Required	Contact screening, frequency selection, amplitude titration, and final program storage
Surface EMG system	Optional validation tool	Optional objective electrophysiological confirmation of paraspinal muscle responses
High-resolution muscle ultrasound	Optional validation tool	Optional visualization of stimulation-induced movement in superficial and deeper paraspinal tissue compartments

Complete technical stimulation parameters and programming specifications required for replication are provided in Table 2B.

In the clinical studies performed to date, MuscleSCS was evaluated using lead systems from a single manufacturer, including an eight-contact percutaneous cylindrical lead and a percutaneously implanted paddle-type lead (12). The main electrode levels were T7/8, T8/9, and T9/10, with the majority located at T8/9. In the later randomized crossover study, octrodes were used. These data suggest that MuscleSCS is technically feasible with different SCS lead types, although programming characteristics may differ between lead geometries.

## 4 Method

### 4.1 Objectives of the method

The objectives of MuscleSCS programming are: (1) to identify low-frequency settings that induce comfortable paraspinally mediated muscle modulation; (2) to select contact combinations that target the clinically relevant painful muscle region; (3) to avoid uncomfortable distal, gluteal, abdominal, or lower-limb activation; (4) to determine an amplitude that produces clear but comfortable modulation; and (5) to integrate the final MuscleSCS program into the patient's overall SCS therapy.

### 4.2 Patient selection

Potential candidates are patients with chronic axial back pain who already have an implanted SCS system and have persistent axial pain despite otherwise adequate SCS therapy. The intended clinical phenotype is characterized at baseline, before MuscleSCS programming, by pain predominantly localized to the paraspinal region together with one or more clinical features suggestive of paraspinal muscular involvement, such as reproducible paraspinal tenderness on palpation, persistent perceived muscle tension or guarding, or pain associated with paraspinal muscle use or loading. These findings are considered supportive clinical features and do not establish the paraspinal musculature as the anatomical source of pain.

There is currently no validated diagnostic gold standard or validated clinical case definition for a “MuscleSCS-responsive muscular pain component.” Accordingly, candidate identification represents clinical phenotyping rather than definitive etiological diagnosis. Baseline phenotyping should be completed before MuscleSCS programming. Subsequent pain relief or perceived muscle relaxation during MuscleSCS is considered a treatment outcome and is not used as a criterion for defining the baseline phenotype.

#### 4.2.1 Contraindications and precautions

MuscleSCS programming should not be initiated in patients with active infection involving the implanted SCS system or overlying tissues, suspected lead or implantable pulse generator malfunction, clinically relevant lead migration or other hardware instability, or inability to provide reliable real-time feedback regarding stimulation-induced sensations and muscle activation. Standard contraindications and safety restrictions regarding the implanted SCS system remain applicable.

Because MuscleSCS intentionally evokes rhythmic paraspinal muscle activity, additional caution is required in patients in whom repetitive trunk-muscle activation may pose a clinical risk. Such conditions should be assessed individually before programming. MuscleSCS-specific evidence is currently insufficient to define a comprehensive set of additional disease-specific absolute contraindications; therefore, these situations should be regarded as clinical precautions rather than established MuscleSCS-specific contraindications unless stimulation is prohibited by the implanted-system labeling or the treating physician determines that evoked muscle activation would be unsafe.

### 4.3 Electrode assessment before programming

Before MuscleSCS programming, the electrode type, rostrocaudal position, laterality, and current SCS programs should be reviewed. If clinically indicated, imaging should be used to confirm electrode position. The anatomical position of the lead is important because contact selection determines which spinal segments and muscle regions are preferentially modulated. In the pilot study, contacts 3&4 and 5&6 most often produced lumbar muscle modulation, whereas contacts 7&8 more frequently involved gluteal muscles, legs, or feet (12).

### 4.4 Step-by-step programming protocol

Table 2A provides a step-by-step programming protocol. In experienced hands, the practical programming procedure is similar to SCS programming and usually requires approximately 15–20 min. The exact duration depends on patient feedback, number of programs tested, lead type, and whether objective confirmation with EMG or ultrasound is performed. The currently evaluated clinical protocol consists of 30 min sessions twice daily. Longer or continuous application has not yet been systematically evaluated and should be investigated in future studies. MuscleSCS and BurstDR are not delivered simultaneously but sequentially, requiring patients to switch between the stimulation programs themselves; however, patients in the previous clinical studies were able to manage this process without difficulty (Figure 1).

**Table 2A:** Step-by-Step MuscleSCS programming protocol.

Step	Action	Expected outcome
1	Place the patient in a comfortable supine or seated position, depending on clinical setting and patient tolerance.	The patient can perceive and describe back muscle modulation reliably.
2	Select a low-frequency MuscleSCS range, typically 4, 6, and 8 Hz. In exploratory settings, 2 Hz may also be tested.	Identification of frequency preference; most patients prefer 6 or 8 Hz, but individual variation exists.
3	When individualized contact selection is required, sequentially screen adjacent bipolar contact pairs (e.g., 1–2, 3–4, 5–6, and 7–8).	Segmental mapping of muscle regions affected by each contact pair.
4	Increase amplitude gradually from the perception threshold to the lowest amplitude producing clearly perceptible, comfortable, rhythmic muscle activity in the intended paraspinal target region. Stop further escalation once adequate target-region activity is achieved or if pain, burning, forceful contraction, cramping, or unwanted non-target activation occurs.	Determination of perception threshold and therapeutic comfort range.
5	Ask the patient to classify each setting as pleasant, neutral, or unpleasant and to indicate the location of the perceived muscle modulation.	Patient-centered selection of candidate programs.
6	Avoid/terminate testing of settings producing: pain or burning, uncomfortable or forceful contraction, cramping or spasm, excessive distal leg activation, abdominal or intercostal activation, or other clinically inappropriate non-target muscle activation. Reduce amplitude first; if the response persists, change the contact pair and/or stimulation setting.	Improved tolerability and safety.
7	Store the selected program. For application according to the previously investigated clinical protocol, MuscleSCS is used for 30 min twice daily. This schedule has not been established as an optimal dosing regimen.	Practical implementation in daily therapy.
8	Reassess pain relief, muscle comfort, adverse sensations, and interaction with conventional SCS at follow-up.	Optimization and long-term integration with SCS therapy.

Technical stimulation parameters required for implementation are provided in Table 2B.

**Figure 1:**
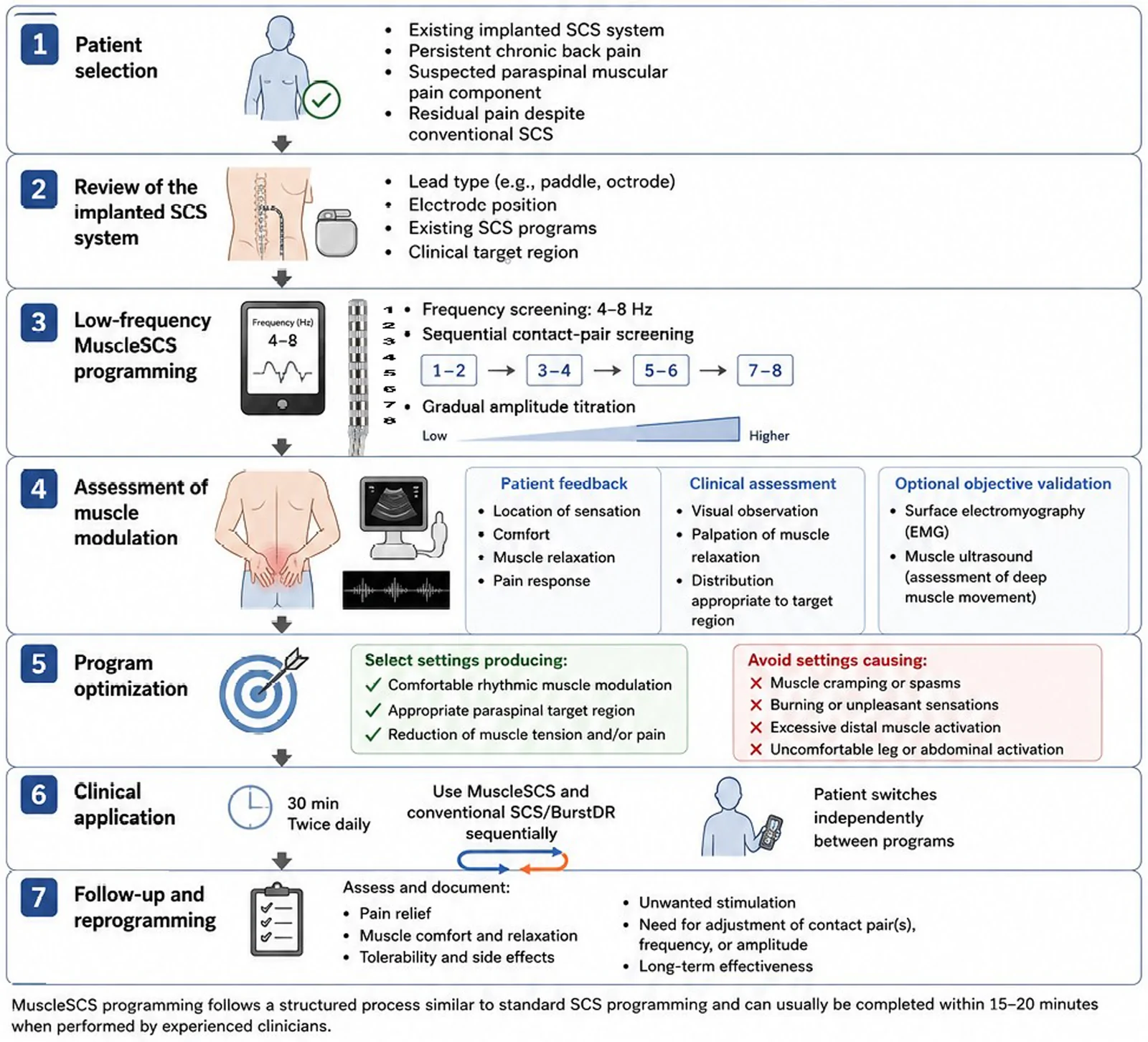
Clinical workflow for MuscleSCS programming, validation, and implementation. MuscleSCS is implemented in selected patients with an existing spinal cord stimulation system and a clinically suspected paraspinal muscular pain component. Following assessment of electrode type, position, and existing stimulation programs, low-frequency stimulation is screened within the clinically evaluated range of 4–8 Hz. Electrode contact pairs are tested sequentially, and stimulation amplitude is gradually increased until comfortable, rhythmic, target-region muscle modulation is achieved. Patient feedback and clinical assessment guide program selection, while surface electromyography and muscle ultrasound may be used for optional objective validation. Settings producing discomfort, cramping, burning sensations, or excessive distal muscle activation are avoided. The selected MuscleSCS program is used sequentially with conventional SCS and is reassessed during follow-up to permit individualized reprogramming. For the complete MuscleSCS stimulation parameter set and technical specifications, see Table 2B.

**Table 2B:** Technical settings.

Parameter	Protocol specification
Pulse waveform	Biphasic rectangular
Pulse width	500 μs
Control mode	Current-controlled
Frequency	2, 4, 6, and 8 Hz sequentially screened; 4–8 Hz evaluated in the previous randomized crossover study; optimal frequency not yet established.
Amplitude	Typically 2.0–8.0 mA; individualized
Configuration	Bipolar; adjacent-contact screening may be used for individualized programming; pragmatic strategy not prospectively validated in the randomized trial.
Polarity	Cathode/anode assignment individualized
Impedance	Electrode impedance should be assessed before programming according to the specifications of the implanted system. In the system evaluated in the previous MuscleSCS studies, impedance values of 50–150 Ω were considered acceptable.
Threshold	Perception threshold → lowest amplitude producing comfortable target-region rhythmic muscle activity

#### 4.4.1 Technical stimulation parameters

To permit independent replication of the previously evaluated MuscleSCS protocol without requiring reconstruction of stimulation settings from earlier publications or proprietary programming knowledge, the essential technical parameters are specified below and summarized in Table 2B. The MuscleSCS protocol evaluated to date was implemented on a single manufacturer's SCS platform using either an eight-contact percutaneous cylindrical lead or a percutaneously implanted paddle-type lead. Stimulation was current-controlled and delivered using biphasic rectangular pulses with a fixed pulse width of 500 μs. Frequencies of 2, 4, 6, and 8 Hz were screened. The subsequent randomized crossover study evaluated frequencies of 4–8 Hz; this range is therefore referred to as the previously studied clinical range and not as an established optimal frequency range. Stimulation amplitude was expressed in mA and individually titrated, typically within a range of approximately 2.0–8.0 mA.

In the initial pilot study, different electrode configurations were explored, whereas the subsequent randomized crossover study used a fixed 3–4 contact configuration to standardize treatment conditions. In the present programming framework, sequential screening of adjacent bipolar contact pairs (e.g., 1–2, 3–4, 5–6, and 7–8) is proposed as a pragmatic strategy to individualize the target-region muscle response. This systematic contact-screening approach has not itself been prospectively validated.

Cathode/anode assignment was individualized according to the distribution and comfort of the evoked muscle response. Amplitude was gradually increased from the perception threshold until clearly perceptible, comfortable, rhythmic target-region muscle activity was achieved. The objective was to identify the lowest effective amplitude producing the desired muscle response without discomfort, cramping, or unwanted distal activation. Electrode impedance was checked before programming, with values between 50 Ω and 150 Ω considered acceptable for the evaluated system.

Thus, pulse waveform and pulse width were fixed protocol parameters, and the low-frequency screening range was predefined. Active contact pair, cathode/anode assignment, and stimulation amplitude were individualized. The protocol has not yet been clinically validated on SCS platforms from other manufacturers. Differences in lead geometry, contact dimensions and spacing, IPG characteristics, and programming architecture may affect stimulation thresholds and field distribution; therefore, transfer to other systems requires device-specific evaluation and validation.

#### 4.4.2 Procedural safety, stopping criteria, and follow-up

Before MuscleSCS programming, the implanted system should be assessed for clinical evidence of hardware dysfunction or instability, and electrode impedance should be checked. MuscleSCS should not be initiated in the presence of active infection involving the implanted system or overlying tissues, suspected clinically relevant lead or IPG malfunction or instability, inability of the patient to provide reliable real-time feedback, or a clinical condition in which stimulation-evoked trunk-muscle activation is considered unsafe. Standard contraindications and safety restrictions applicable to the implanted SCS system remain applicable.

Initial titration should be performed with the patient in a comfortable, stable supine position that permits continuous communication and observation of the stimulation-evoked response. Amplitude is increased gradually from the perception threshold. The programming objective is the lowest effective amplitude producing comfortable rhythmic muscle activity in the intended paraspinal target region, without cramping, pain, or unwanted non-target activation.

No quantitative maximum tolerable muscle activation or MuscleSCS-specific maximum stimulation amplitude has been established. Therefore, the upper titration limit is clinically defined by patient tolerance and the distribution and character of the evoked response. Further amplitude escalation should be stopped once adequate target-region muscle activity is obtained or if stimulation produces pain, burning or other unpleasant sensations, forceful or uncomfortable contraction, muscle cramping or spasm, or unwanted activation outside the intended paraspinal target region.

Distal leg, abdominal, or intercostal activation is considered non-target stimulation. If such activation occurs, amplitude should first be reduced. If non-target activation persists or prevents adequate target-region stimulation, another contact pair and/or stimulation setting should be selected. Settings producing persistent uncomfortable or clinically inappropriate non-target activation should not be stored for therapeutic use.

Programming should be terminated if clinically relevant adverse symptoms occur, reliable patient feedback cannot be maintained, or device integrity or impedance findings raise concern regarding proper system function. Adverse sensations and unintended motor responses should be documented during programming. Patients should be instructed to discontinue the MuscleSCS program and contact the treating SCS center if new painful stimulation, persistent cramping, unexpected non-target muscle activation, new neurological symptoms, or other concerning effects occur during home use.

Follow-up should reassess pain response, perceived muscle comfort, stimulation distribution, tolerability, adverse effects, and interaction with the patient's conventional SCS program. Reprogramming should be considered in cases of insufficient or decreasing therapeutic benefit, uncomfortable stimulation, altered distribution of muscle activation, new unwanted stimulation, or suspected changes in device function. A validated MuscleSCS-specific follow-up interval has not yet been established; follow-up should therefore be integrated into the clinical schedule of the treating SCS center, with earlier reassessment when clinically indicated.

At present, no objectively validated MuscleSCS-specific biomarker or quantitative physiological threshold defines the therapeutic stimulation range; therefore, programming combines technically standardized stimulation parameters with individualized clinical assessment of target-region muscle activity, comfort, and avoidance of adverse or non-target responses.

### 4.5 Frequency selection

The initial MuscleSCS pilot study systematically evaluated stimulation frequencies of 2, 4, 6, and 8 Hz (12). Within this range, 6 Hz was most frequently preferred in patients with percutaneous paddle-type leads, whereas 8 Hz was most frequently preferred in patients with octrodes. The subsequent randomized crossover study using octrodes evaluated MuscleSCS within a frequency range of 4–8 Hz (32). Accordingly, 4–8 Hz represents the previously studied clinical range rather than an established or definitive optimal frequency range.

For the standardized protocol described here, frequencies of 2, 4, 6, and 8 Hz are sequentially screened and the frequency producing the most comfortable and appropriate target-region muscle modulation is selected individually. Current evidence is insufficient to establish an universally optimal MuscleSCS frequency. Emerging observations suggest that useful frequencies may extend beyond the range investigated in the published studies; however, these findings require prospective validation. Future studies should systematically investigate broader frequency ranges and determine whether optimal frequency depends on lead geometry, stimulation location, or individual patient characteristics.

### 4.6 Contact selection

Contact selection determines the region of muscle modulation (12). In the pilot study, contact pairs 3&4 and 5&6 frequently produced lumbar muscle modulation, whereas 1&2 more often involved thoracic regions and 7&8 more often involved gluteal, leg, or foot areas. Contacts 3&4 were most often preferred overall. Therefore, screening should generally include all contact pairs, but contacts 3&4 and 5&6 are useful starting points when the therapeutic target is the lumbar paraspinal musculature.

### 4.7 Amplitude titration

Amplitude should be increased gradually and always remain within the patient's comfort range. The goal is not to maximize visible movement. The ideal amplitude is the lowest level that generates clearly perceptible, comfortable, target-region muscle modulation and pain relief without discomfort or unwanted distal activation. If the patient reports burning, sharp pain, excessive cramping, abdominal spread, leg activation, or unpleasant internal sensation, amplitude should be reduced and another contact or frequency should be tested.

### 4.8 Recommended clinical use

Regular rather than sporadic application of MuscleSCS is proposed as a potential treatment strategy; however, the superiority of regular application has not yet been established. This recommendation is consistent with its mechanotransductive rationale: tissue adaptation requires repeated mechanical signals over time. In earlier clinical work, MuscleSCS was applied in defined daily sessions; future protocols may evaluate longer or continuous daily use. Whether longer or continuous application provides additional clinical benefit remains to be investigated. In clinical practice, the treatment plan should be individualized according to patient tolerance, pain pattern, device capacity, and interaction with other SCS programs (Figure 1).

## 5 Previous clinical evidence and objective confirmation of MuscleSCS effects

The present Methods article does not report a new clinical dataset and therefore does not include a new statistical analysis. The evidence summarized in this section derives from previously published clinical investigations of MuscleSCS. Accordingly, the term “validation” is used only where an objectively observed stimulation-induced effect was confirmed by an independent measurement method and should not be interpreted as comprehensive validation of the clinical efficacy, optimal programming parameters, or proposed biological mechanisms of MuscleSCS.

### 5.1 Clinical feasibility

The first clinical pilot study included 17 patients with chronic back pain and implanted SCS systems (12). Eleven patients had percutaneous paddle leads and six had octrodes. Low-frequency MuscleSCS was feasible in all patients. Patients evaluated the resulting muscle modulation by subjective comfort and pain relief, while clinicians assessed visible and palpable muscle responses. The study showed that low-frequency programs delivered through SCS electrodes can generate comfortable paraspinally mediated muscle modulation in chronic back pain patients.

### 5.2 Electrophysiological confirmation

Surface EMG is not required for routine MuscleSCS programming and should be regarded as an optional objective validation or research method. In the pilot study, sEMG was performed in three patients to confirm that clinically observed rhythmic movements corresponded to genuine muscle activity. Four bipolar surface electrode pairs were positioned over the upper thoracic, lower thoracic, upper lumbar, and lower lumbar paraspinal regions (12). Myogenic potentials were recorded in regions corresponding to the visually or palpably activated muscles. These measurements provided electrophysiological confirmation of MuscleSCS-induced muscle activity but were not used to determine routine programming parameters or patient eligibility. A standardized MuscleSCS-specific sEMG acquisition and analysis protocol has not yet been established and should be developed in future prospective studies.

### 5.3 Sonographic confirmation

Ultrasound was used in the pilot study as an optional exploratory validation method to assess the depth of stimulation-induced paraspinal tissue movement. The objective was not to identify individual deep paraspinal muscles or to perform muscle-specific anatomical segmentation. Ultrasound demonstrated rhythmic tissue displacement corresponding to clinically observed and EMG-confirmed muscle activity, including movement at depths of approximately 4 cm and greater (12). These observations indicate that MuscleSCS-induced mechanical activity can extend into deeper paraspinal tissue compartments. However, ultrasound alone does not identify the specific muscle involved and does not establish the underlying neural recruitment mechanism. Standardized muscle-specific ultrasound protocols and anatomical characterization should be developed in future studies.

### 5.4 Previously reported clinical outcomes

A prospective, single-center, single-blinded, randomized crossover study subsequently evaluated MuscleSCS in patients with chronic low back pain previously treated with BurstDR SCS, using octrodes exclusively (32). The study compared BurstDR alone, MuscleSCS alone, and combined BurstDR plus MuscleSCS. The combined application produced significantly better pain outcomes than BurstDR alone (*P* = 0.032), and 71.5% of patients experienced some degree of pain improvement with additional MuscleSCS. This percentage represents patients reporting pain improvement and was not based on a predefined responder threshold. PDI scores did not show a corresponding significant improvement.

### 5.5 Treatment schedule and duration

In the previous clinical investigations of MuscleSCS, stimulation was applied for 30 min twice daily. This schedule represents the regimen used in those studies and should not be interpreted as an optimized treatment dose. The optimal duration of individual MuscleSCS sessions, number of daily sessions, cumulative daily stimulation time, and potential role of longer or continuous stimulation have not been systematically evaluated. No MuscleSCS-specific dose-response relationship has currently been established. Accordingly, recommendations regarding treatment frequency and duration remain empirical and should be evaluated in prospective comparative dosing studies.

## 6 Anticipated results

When correctly programmed, MuscleSCS should produce comfortable, rhythmic modulation of the target paraspinal muscle region. The patient should perceive the effect as pleasant, relaxing, and pain relieving. The clinician may observe or palpate rhythmic muscle movement. Objective confirmation can be obtained with EMG and ultrasound when required.

The most clinically useful results are usually obtained with settings that produce gentle but clear paraspinal modulation, avoid distal spread, and can be tolerated for regular daily use. A setting that produces maximal visible movement is not necessarily the best therapeutic setting (Table 3).

**Table 3:** Expected findings, clinical interpretation, and troubleshooting during MuscleSCS programming.

Expected finding	Clinical meaning	Troubleshooting if absent
Comfortable target-region muscle modulation	Successful stimulation-evoked target-region muscle activity; underlying neural recruitment pathway undetermined.	Increase amplitude slowly; test adjacent contacts; test 4, 6, and 8 Hz
Pain reduction during or after use	Clinical analgesic response to MuscleSCS; mechanism and anatomical source of pain cannot be determined from treatment response alone.	Optimize contact region and stimulation parameters; assess response over follow-up; consider interaction with conventional SCS therapy.
Unwanted gluteal or leg activation	Contact pair too caudal or excessive spread	Use more rostral contacts or reduce amplitude
Burning or unpleasant sensation	Excessive amplitude or unsuitable contact/frequency	Reduce amplitude; switch frequency; choose another contact pair
No visible but perceived deep modulation	Deep muscle recruitment without superficial movement	Consider ultrasound or EMG validation if clinically important

## 7 Advantages, limitations, pitfalls, and troubleshooting

### 7.1 Advantages

The major advantage of MuscleSCS is that it uses the existing SCS hardware. No additional peripheral nerve lead, muscle electrode, or separate implant is required. The method can therefore be added to the therapeutic portfolio of selected SCS patients through programming. It can complement established SCS waveforms such as BurstDR or tonic SCS and may be particularly valuable when residual pain is driven by paraspinal muscle tension or muscle nociception.

A second advantage is anatomical reach. Depending on lead position and contact selection, MuscleSCS can modulate broad regions of the back, including thoracic, lumbar, gluteal, and deep paraspinal muscle compartments. This distinguishes it from methods that target a single peripheral nerve or a single muscle group.

A third advantage is programming simplicity. The protocol resembles standard SCS programming and can usually be completed within 15–20 min by experienced clinicians. The method is therefore clinically practical and does not require a fundamentally new implantation technique.

### 7.2 Limitations

Current limitations include the still limited clinical evidence base, small sample sizes in the initial feasibility work, lack of direct molecular biomarkers of mechanotransduction, and the need for prospective studies that evaluate long-term outcomes, optimal use schedules, and interaction with different SCS waveforms. The exact anatomical structures recruited by MuscleSCS may vary across lead types, electrode positions, spinal anatomy, scar tissue, and patient-specific thresholds.

A further limitation is that MuscleSCS has not yet been directly shown to alter macrophage phenotype, focal adhesion signaling, YAP/TAZ activation, nociceptor sensitization, or muscle inflammatory markers in humans (33). The mechanotransductive model is biologically plausible and supported indirectly by findings from the broader mechanobiology literature (34, 35), but it remains a hypothesis that should be tested specifically in MSCS.

The currently available clinical evidence should therefore be considered preliminary and does not establish long-term efficacy, comparative effectiveness, or generalizability of MuscleSCS beyond the populations and stimulation systems studied to date. The present Methods Article is intended to standardize the intervention sufficiently for prospective independent evaluation rather than to establish its clinical efficacy.

### 7.3 Pitfalls and artifacts

Potential pitfalls include confusing uncomfortable motor activation with therapeutic muscle modulation, selecting settings based only on visible movement rather than patient comfort, using the method sporadically despite the need for repeated mechanobiological input, or describing the method in a way that implies a simple comfort procedure rather than a neuromodulative therapy. Unwanted distal activation, excessive cramping, and unpleasant sensory phenomena can usually be avoided by reducing amplitude, changing contact pairs, or selecting another frequency.

## 8 Discussion

The methodological value of MuscleSCS lies in the reproducibility of its programming workflow and in the possibility of validating the induced muscle response through clinical assessment, EMG, ultrasound, and future quantitative biomechanical measures. At present, MuscleSCS should be considered a combined programming approach in appropriately selected patients with an existing SCS system and not an independent indication for SCS implantation.

### 8.1 MuscleSCS addresses an unmet biological target in chronic back pain

Spinal cord stimulation (SCS) is an established neuromodulation therapy for selected chronic neuropathic pain conditions, including persistent spinal pain syndrome type 2 (PSPS-T2) (36, 37), with studies reporting improvements in pain, function, and quality of life in appropriately selected patients (36, 38, 39). However, outcomes in chronic low-back pain are heterogeneous and depend on factors including the underlying indication and pain phenotype, patient selection, stimulation paradigm, comparator, and study design. Pain should be interpreted within the context of the specific patient population and therapeutic approach.

Notably, the 2023 Cochrane review concluded that placebo-controlled evidence probably indicates little or no benefit of SCS for the outcomes evaluated in the studied populations. This conclusion should be considered alongside the broader and heterogeneous SCS literature and interpreted in the context of differences in patient selection, indication, stimulation paradigm, comparator, and study design.

However, clinically relevant residual axial low back pain may persist despite successful SCS therapy and many patients experience persistent back pain despite successful implantation and adequate control of radicular symptoms (40, 41). This suggests that additional biological pain generators remain insufficiently addressed (42).

One important candidate is the paraspinal musculature. Chronic low back pain has been associated with impaired motor control, altered paraspinal muscle morphology, fatty infiltration, fibrosis, and changes in muscle composition and function (20–22, 26, 27, 43). Rather than being merely secondary consequences of pain, these alterations may create a pathological tissue environment that sustains nociceptive input (44). Skeletal muscle may therefore represent a biologically active contributor to chronic pain persistence (45).

MuscleSCS was developed to address this therapeutic gap. Rather than replacing conventional SCS, it extends the function of the existing implanted system by using low-frequency epidural stimulation to recruit spinal motor pathways and generate rhythmic paraspinal muscle activity. Because no additional hardware is required, MuscleSCS represents a novel biological application of established neuromodulation technology.

Its innovation lies not in new technology but in a new therapeutic concept. Whereas conventional SCS primarily targets neuronal pain-processing pathways, MuscleSCS proposes that the same implanted system may additionally modulate chronically dysfunctional paraspinal musculature. This broadens the therapeutic scope of neuromodulation and forms the basis of dual-target neuromodulation.

### 8.2 The concept of dual-target neuromodulation

The central concept of MuscleSCS is that chronic back pain may be maintained by two biologically distinct but interconnected systems: the neuronal pain-processing network, the established target of conventional spinal cord stimulation (SCS), and chronically dysfunctional paraspinal musculature, which may become an independent source of persistent nociceptive input through structural remodeling, chronic inflammation, altered mechanical properties, and impaired tissue homeostasis (46).

Conventional SCS modulates neuronal pain processing, whereas MuscleSCS complements these mechanisms by addressing the muscular component through spinally mediated activation of motor pathways. Rather than replacing conventional SCS, it integrates two complementary biological targets within a single implanted neuromodulation system.

MuscleSCS should therefore be regarded not as an additional stimulation program but as a concept of dual-target neuromodulation, combining established neural neuromodulation with the proposed mechanobiological modulation of chronically dysfunctional skeletal muscle. The neural axis targets central pain processing, whereas the muscular axis aims to influence peripheral tissue biology through spinally mediated rhythmic muscle activation.

Importantly, the therapeutic target is the spinal motor system rather than skeletal muscle itself. Low-frequency epidural stimulation recruits spinal motor pathways, generating rhythmic paraspinal muscle activity and mechanical tissue loading rather than directly stimulating muscle tissue. MuscleSCS therefore represents spinally mediated muscle modulation and is distinct from conventional neuromuscular electrical stimulation, which primarily aims to strengthen muscle or restore motor function (Figure 2).

**Figure 2:**
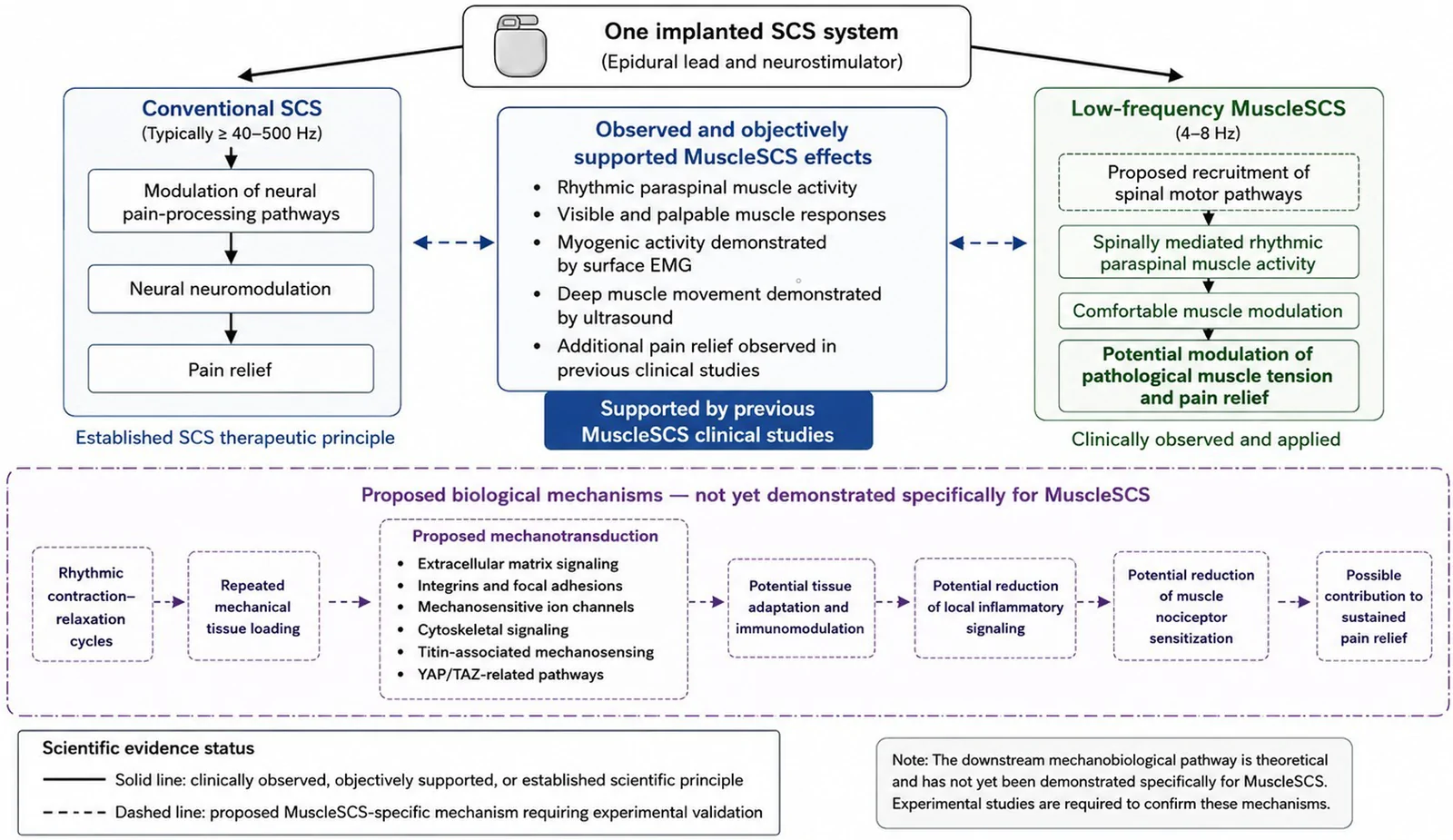
Dual-target neuromodulation and the hypothesis-driven mechanobiological model of MuscleSCS. A single implanted spinal cord stimulation system may provide two complementary therapeutic approaches. Conventional SCS targets neuronal pain-processing pathways through established mechanisms of neural neuromodulation. MuscleSCS uses low-frequency epidural stimulation to generate spinally mediated rhythmic paraspinal muscle activity. The generation of paraspinal muscle responses has been clinically observed and objectively supported by surface electromyography and muscle ultrasound, while previous clinical studies have demonstrated the feasibility of the method and additional pain relief in selected patients. The downstream mechanobiological pathway remains hypothetical. Repeated contraction–relaxation cycles are proposed to generate controlled mechanical tissue loading that may activate mechanotransductive pathways involving the extracellular matrix, integrins, focal adhesions, mechanosensitive ion channels, cytoskeletal signaling, titin-associated mechanosensing, and YAP/TAZ-related pathways. Potential subsequent effects on tissue homeostasis, inflammatory signaling, immune-cell behavior, and muscle nociceptor sensitization have not yet been demonstrated specifically for MuscleSCS and require experimental validation. Solid lines indicate clinically observed findings, objectively supported effects, or established scientific principles; dashed lines indicate proposed MuscleSCS-specific mechanisms. For the complete MuscleSCS stimulation parameter set and technical specifications, see Table 2B.

### 8.3 Mechanotransduction as a proposed biological framework for MuscleSCS

The mechanisms discussed in this section represent hypothesis-generating extrapolations from established skeletal-muscle mechanobiology and have not been directly demonstrated in MuscleSCS. The biological rationale of MuscleSCS is based on mechanotransduction (34). Mechanotransduction is the process by which cells convert mechanical stimuli into biochemical signals (47–49). In skeletal muscle, mechanical deformation is sensed by multiple structures, including the extracellular matrix, integrins, focal adhesions, stretch-activated ion channels, the cytoskeleton, sarcomeric proteins such as titin, and nuclear mechanotransduction pathways involving YAP (Yes-associated protein) and TAZ (Transcriptional co-activator with PDZ-binding motif, also known as WW domain-containing transcription regulator 1 [WWTR1]) (33, 35). These pathways can influence protein synthesis, inflammatory signaling, cell survival, apoptosis, autophagy, tissue remodeling, and regeneration (50–52).

#### 8.3.1 From spinally mediated musclemodulation to mechanical tissue signaling

MuscleSCS is proposed to engage spinal motor pathways, including possible alpha motoneuron-mediated motor output, thereby generating rhythmic paraspinal muscle activity. This produces repeated cycles of contraction and relaxation. Each cycle generates mechanical strain, tissue deformation, fluid movement, shear stress, and cytoskeletal loading within the muscle and its connective tissue matrix (53, 54). These physical signals are not biologically inert (55). They can be transduced into molecular responses through mechanosensitive structures in muscle fibers, satellite cells, fibroblasts, vascular cells, immune cells, and afferent nerve-associated tissues (56).

This mechanobiological model is proposed to be central to MuscleSCS (57). The aim is not to produce exercise-like strengthening. Instead, the aim is to provide controlled rhythmic mechanical input to chronically dysfunctional muscle tissue, with the intended effects of reducing pathological tone, and potentially shifting the local tissue environment toward repair and reduced nociceptive sensitization.

#### 8.3.2 Focal adhesions, integrins, and the cytoskeleton

Focal adhesions are specialized sites where cells connect the extracellular matrix to the actin cytoskeleton through integrins and associated proteins such as focal adhesion kinase, vinculin, talin, and paxillin (52, 58). They are both structural anchors and signaling hubs. Mechanical forces transmitted through the extracellular matrix can alter focal adhesion conformation, kinase activity, cytoskeletal tension, and downstream signaling. In skeletal muscle, these processes are relevant because chronic abnormal loading, disuse, spasm, and fibrosis can alter the mechanical microenvironment of the tissue (59).

By inducing repeated low-frequency mechanical deformation, MuscleSCS may provide a controlled mechanobiological stimulus to these signaling hubs. This may be particularly relevant in chronic back pain patients whose paraspinal muscles are exposed to long-lasting protective contraction, altered loading, and tissue remodeling (60).

#### 8.3.3 Mechanosensitive ion channels and calcium signaling

Mechanosensitive ion channels, including Piezo and transient receptor potential channel families, can convert membrane deformation into ionic currents and intracellular calcium signaling (52, 61). Calcium is a central second messenger in muscle adaptation, inflammatory signaling, mitochondrial function, and gene expression. The mechanotransduction literature suggests that focal adhesion signaling and mechanosensitive ion channels do not operate in isolation but interact as parts of broader mechanobiological networks (51, 58, 62). This supports the hypothesis that MuscleSCS-induced rhythmic tissue deformation may activate multiple mechanosensitive pathways simultaneously.

#### 8.3.4 Titin, sarcomeric mechanosensing, and YAP/TAZ

Skeletal muscle also contains intrinsic sarcomeric mechanosensors (50). Titin acts as a passive tension sensor and is involved in signaling pathways related to protein turnover and cellular adaptation (19). Z-disc and M-line proteins participate in the transformation of mechanical strain into biochemical responses. At the nuclear and transcriptional level, YAP and TAZ act as mechanotransduction relays that respond to cytoskeletal tension, extracellular matrix stiffness, and cell shape (35, 51). These pathways are involved in tissue growth, regeneration, and pathological remodeling. Although direct activation of these pathways by MuscleSCS has not yet been demonstrated, they provide a plausible biological framework for future mechanistic studies.

#### 8.3.5 Mechanical immunomodulation and macrophage phenotype

Mechanical loading of skeletal muscle can influence immune-cell behavior (47, 63). Following muscle injury or chronic tissue stress, macrophages contribute dynamically to inflammatory regulation and tissue repair. Although macrophage responses have traditionally been described using M1-like pro-inflammatory and M2-like reparative phenotypes, current evidence supports a broader continuum of context-dependent activation states rather than a strict M1/M2 dichotomy. MuscleSCS-induced mechanical modulation may potentially influence this local immune environment (64); however, such effects have not yet been demonstrated specifically for MuscleSCS.

The mechanotransductive model is biologically plausible and supported indirectly by findings from the broader mechanobiology literature (65–67), but remains to be tested specifically in MuscleSCS. Repetitive spinally mediated muscle modulation may act as an internal, programmable form of mechanical loading, potentially promoting an anti-inflammatory and reparative microenvironment in chronically painful paraspinal muscles. This hypothesis requires direct experimental validation but is consistent with current mechanobiological concepts.

#### 8.3.6 Nociceptor sensitization and pain relief

Muscle nociceptors can be sensitized by inflammatory mediators and neurotrophic factors (14, 15, 19). Prolonged inflammatory signaling can lower nociceptor thresholds, increase afferent firing, and contribute to peripheral and central sensitization. Experimental work has shown that persistent skeletal muscle inflammation can increase the density of neuropeptide-containing nerve endings, while nerve growth factor and substance *P* contribute to enhanced nociceptive signaling (18, 19). If MuscleSCS reduces pathological tone and shifts the local tissue environment away from chronic inflammation, it may reduce nociceptor sensitization and thereby contribute to pain relief.

This model links the clinical observation of pain reduction with a plausible tissue-level mechanism: spinally mediated modulation may reduce muscle tone and generate rhythmic mechanotransductive signaling; mechanotransductive signaling may influence immune-cell behavior and tissue repair; reduced inflammatory signaling may reduce nociceptor sensitization; and reduced nociceptor input may decrease chronic back pain (Figure 2).

### 8.4 Established evidence and biological hypothesis

A major objective of this manuscript is to distinguish established scientific evidence from biological hypotheses. This distinction is essential for evaluating the conceptual framework of MuscleSCS.

Several components of the proposed model are supported by established evidence. The neurophysiological mechanisms of conventional spinal cord stimulation are well characterized and form the basis of modern neuromodulation. Chronic low back pain is consistently associated with structural and biological abnormalities of the paraspinal muscles, including fibrosis, chronic inflammation, extracellular matrix remodeling, altered muscle activation, including persistent guarding or increased muscle tone in selected patients, and altered motor control (68). Mechanobiology has demonstrated that mechanical loading regulates cellular behavior, extracellular matrix turnover, inflammatory signaling, vascular adaptation, and tissue regeneration. Furthermore, low-frequency epidural stimulation produces objectively verifiable rhythmic paraspinal muscle activity, consistent with recruitment of spinal motor pathways. Together, these findings provide the scientific foundation for MuscleSCS. In contrast, the integration of these mechanisms into a therapeutic pathway specific to MuscleSCS remains hypothetical. It is unknown whether rhythmic spinally mediated muscle activation induces mechanotransduction, modifies extracellular matrix remodeling, reduces inflammation, restores tissue homeostasis, or whether these biological effects directly contribute to the clinical benefits observed with MuscleSCS. These mechanisms should therefore be regarded as biologically plausible hypotheses rather than experimentally confirmed mechanisms of action. Accordingly, MuscleSCS should be viewed as a hypothesis-driven biological concept rather than a completed mechanistic model. Its value lies in integrating established knowledge from neurophysiology, skeletal muscle biology, mechanobiology, and pain neuroscience into a coherent framework that generates clear, experimentally testable hypotheses. Importantly, it has not yet been established whether the magnitude, spatial distribution, duration, and type of mechanical deformation generated by MuscleSCS are sufficient to activate these mechanotransductive pathways in chronically painful human paraspinal muscle. Establishing this link will require direct biomechanical and molecular investigation.

Mechanotransduction represents one plausible explanation for the observed clinical effects of MuscleSCS but should not be considered the exclusive mechanism. Analgesia associated with rhythmic paraspinal muscle activation may also involve altered afferent and proprioceptive input, segmental reflex modulation, changes in local muscle perfusion, sensory or expectancy effects, and interactions with conventional SCS mechanisms. The relative contribution of these mechanisms has not yet been determined and requires further investigation.

### 8.5 Scientific implications

The conceptual framework proposed in this manuscript extends beyond the introduction of a new stimulation program.

Its principal contribution is not the recruitment of spinal motor circuits by epidural stimulation itself, which is well established, but the application of this principle as an adjunctive muscle-directed strategy for chronic back pain using an existing SCS system, together with a reproducible programming framework and a proposed mechanobiological rationale.

Traditionally, SCS has been viewed as a therapy that modulates neuronal activity within pain-processing pathways. MuscleSCS proposes that the same implanted neuromodulation system may additionally influence peripheral biological processes through recruitment of spinal motor circuits, thereby broadening the therapeutic scope of SCS without changing its established principles.

Importantly, MuscleSCS does not require new hardware, electrodes, or implantation techniques. Conventional SCS systems are used, but low-frequency epidural stimulation is specifically applied to generate spinally mediated rhythmic paraspinal muscle activity, producing rhythmic contraction of the paraspinal muscles. The intervention therefore extends beyond modulation of neuronal signaling and may also modify the biological environment of chronically dysfunctional skeletal muscle.

Accordingly, the implanted SCS system can be viewed not only as a neuromodulation device but also as a programmable interface that generates stimulation-evoked rhythmic mechanical activity in paraspinal muscle. This activity should not be assumed to reproduce physiological motor-unit recruitment, force distribution, or normal voluntary contraction patterns.

Electrical stimulation serves as the trigger for spinal motor output, while the resulting mechanical loading is proposed to initiate downstream biological responses. The therapeutic focus therefore shifts from electrical stimulation itself to the potential biological consequences of stimulation-evoked rhythmic muscle activity. MuscleSCS should not be regarded as an alternative to conventional SCS, BurstDR™, high-frequency stimulation (69, 70), dorsal root ganglion stimulation (71–74), or restorative multifidus stimulation (24, 25). Rather, it complements established neuromodulation by targeting a peripheral biological component of chronic back pain and therefore expands, rather than competes with, existing therapies.

The proposed terminology reflects this concept. MuscleSCS is described as spinally mediated muscle modulation, emphasizing activation of spinal motor pathways rather than direct electrical stimulation of muscle tissue. Likewise, dual-target neuromodulation defines the central innovation: a single implanted system simultaneously addressing two biologically distinct therapeutic targets—neuronal pain-processing pathways and chronically dysfunctional paraspinal muscle. This terminology provides a consistent framework for future scientific investigation.

### 8.6 Future directions

The biological model proposed for MuscleSCS generates several experimentally testable hypotheses. Future studies should determine whether repeated spinally mediated muscle activation induces measurable biological changes in chronically dysfunctional paraspinal muscle and whether these changes contribute to sustained clinical improvement. Emphasis should be placed on objective biological endpoints rather than clinical outcomes alone.

Potential approaches include quantitative magnetic resonance imaging, ultrasound elastography (75), electromyography, and molecular analyses of inflammatory mediators (76), extracellular matrix composition (55), mechanotransduction pathways (77), and muscle-derived biomarkers (78). Studies should also evaluate changes in tissue stiffness, extracellular matrix organization (79), inflammatory signaling (80), and other markers of muscle homeostasis, and correlate these findings with clinical outcomes to clarify the proposed mechanobiological mechanisms.

Future research should also identify biological markers that support individualized neuromodulation. Objective measures of muscle quality, tissue stiffness, inflammatory activity, and mechanobiological adaptation could complement symptom-based programming and promote a more biologically informed approach to neuromodulation. Although these applications remain proposed, they represent important directions for future translational research.

## 9 Conclusion

MuscleSCS should not primarily be regarded as another stimulation program within spinal cord stimulation. Rather, it represents a conceptual extension of neuromodulation in which established neural neuromodulation is complemented by chronically dysfunctional paraspinal muscle as a proposed additional therapeutic target. Conventional SCS effectively addresses the neuronal component through established mechanisms of neural neuromodulation. MuscleSCS offers an additional therapeutic axis in which spinally mediated rhythmic muscle activation may influence the biological condition of skeletal muscle through mechanobiological processes.

The concept presented in this manuscript is firmly rooted in established evidence regarding spinal cord stimulation, skeletal muscle biology, and mechanobiology. At the same time, it clearly distinguishes these established principles from the proposed biological mechanisms specific to MuscleSCS. While the downstream mechanobiological effects remain hypothetical and require experimental validation, the overall framework provides a coherent and scientifically plausible model for future investigation.

The principal contribution of MuscleSCS is both methodological and conceptual. Methodologically, it provides a standardized programming framework intended to facilitate reproducible spinally mediated paraspinal muscle modulation. Its reproducibility and generalizability require prospective evaluation and independent external replication.

Conceptually, it introduces dual-target neuromodulation as a framework in which implanted spinal cord stimulation systems may influence both neuronal pain processing and the peripheral biological substrate of chronic pain. If future translational and clinical studies confirm the proposed mechanisms, this concept may broaden the therapeutic scope of neuromodulation beyond the nervous system alone and may establish a new biological perspective for the treatment of chronic back pain.

## Data Availability

The raw data supporting the conclusions of this article will be made available by the authors, without undue reservation.
